# Supplementary material for: Training a spiking neuronal network model of visual-motor cortex to play a virtual racket-ball game using reinforcement learning
Source: PLoS One. 2022 May 11;17(5):e0265808. doi: 10.1371/journal.pone.0265808 (PMC9094569; doi:10.1371/journal.pone.0265808)
Supplement: S1 File — (DOCX) [file pone.0265808.s001.docx]

### **Supplementary Material**


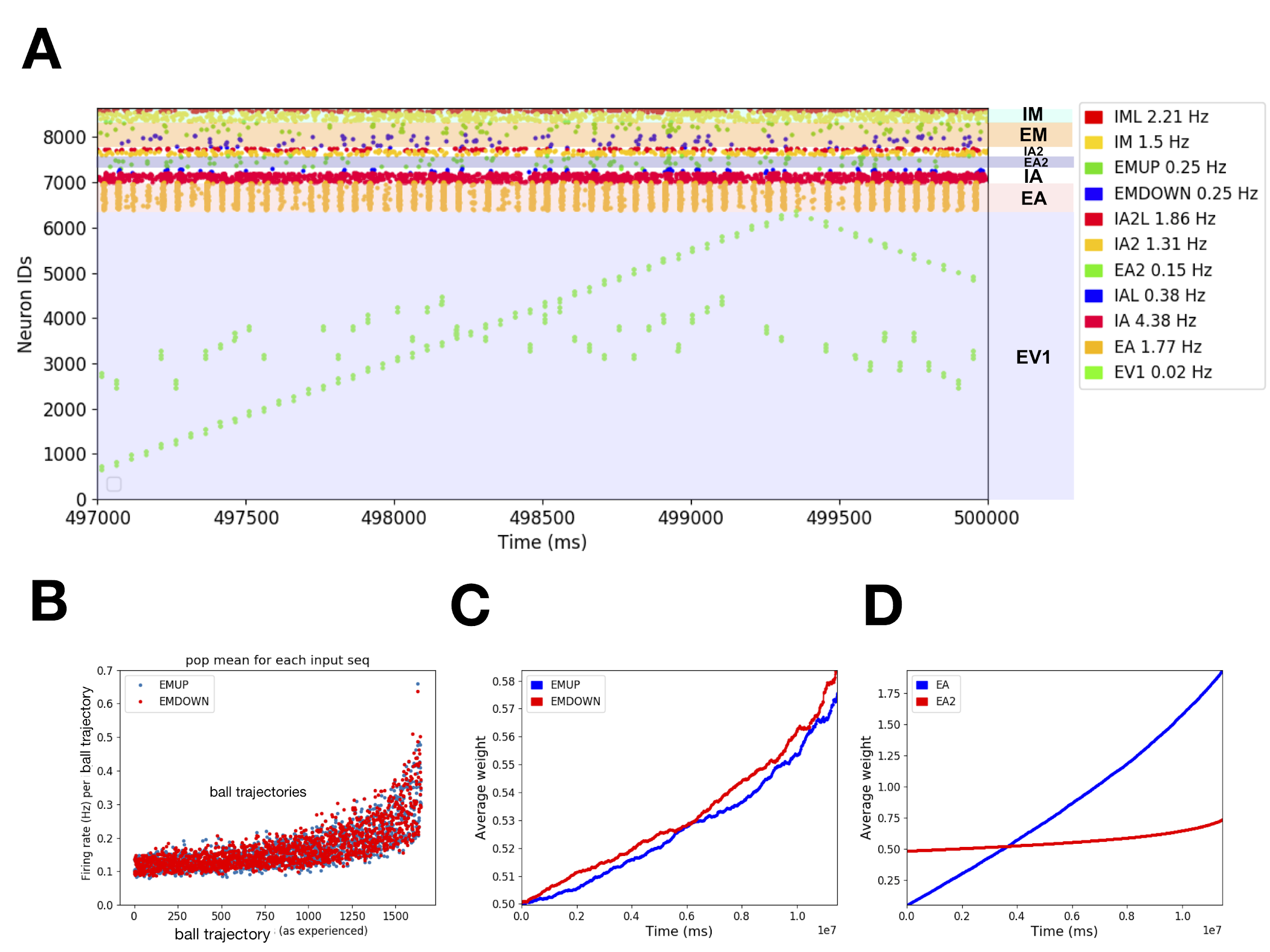


**Supplementary Figure 1:** **A)** Raster plot of different populations of neurons during a training episode (vertical axis is neuron identity and horizontal axis is time; each dot represents a single action potential from an individual neuron). **B)** Firing rates of excitatory motor neuron populations EMUP and EMDOWN in the feedforward model increase over the course of training. The firing rates were binned for ball trajectories (beginning when the ball is at the extreme left side of the court and ends when the ball hits or misses the racket on the right side of the court). **C)** Average weight change of synaptic inputs onto EMUP and EMDOWN sampled over 23 training episodes tends to increase with learning, **D)** Same as in **C** for EA and EA2 populations.

**Training the recurrent model to learn visuo-motor behavior using *retrograde targeted RL***

In contrast to the feedforward model, where STDP-RL mechanism was included only at the synapses onto the motor neurons, in the recurrent model, STDP-RL mechanism was also included at the synapses onto the association neurons, EA and EA2. Because these synapses were not directly involved in action generation, we used a special rule for reinforcement learning that we termed as *retrograde targeted RL* in which the synapses away from the motor areas get partial reward or punishment depending on the ‘critic’ value. Next, similar to the feedforward model, we tuned the parameters of the recurrent model to ensure reliable transmission of neural activity across the modeled areas without causing hyperexcitability or depolarization-block (see raster plot in **Supplementary Figure 1A**).

We trained the recurrent network model to play a bouncing ball game for 23 episodes. During training, both EMUP and EMDOWN neurons in the recurrent model were sparsely active (~0.08-0.15 Hz) at the beginning and later evolved to higher yet still sparse firing rates (~0.2-0.5 Hz) as shown in **Supplementary Figure 1B**. These firing rates were computed for the duration of full ball trajectories from the left side of the court to the right side and show that during training the model experienced 1600 ball trajectories/ input patterns. The increase in firing rates of motor neurons resulted from increase in the synaptic weights of the connections onto EMUP and EMDOWN neurons as well as increase in the synaptic weights of the recurrent and feedback connections onto EA and EA2 neurons as the average weight change of these populations is shown in **Supplementary Figure 1B**. The net increase in average weights of EMUP and EMDOWN neurons was about 16%, whereas the net increase in average weights of EA and EA2 neurons was 1800% and 40% respectively.

In **Figure 3**, we saw that the performance of the feedforward model clearly improved over repeated training episodes. Although we saw increased performance across the first few training episodes of the recurrent model with IRP (**Supplementary Figure 2A and B**), the performance fluctuated over later training episodes (**Supplementary Figure 2A and B**). Even the best performance (0.6) was not as good as the performance of the feedforward model (0.94), however we clearly observed some learning. We also noticed that the recurrent model learned more rapidly than the feedforward model as the recurrent model’s performance improved to 0.5 only after 4 training episodes (and fewer ball trajectories as each action timestep was 50 ms) as compared to 8 training episodes (and more ball trajectories as each action timestep was 20 ms) for the feedforward model. Most of the performance features during training episodes (**Supplementary Figure 2C-E and Supplementary Movie 9-12**) were similar to what we observed for the feedforward model (**Figure 3C-F**) and are described below.

During 23 episodes of training, overall the recurrent model experienced 46 spatially unique ball trajectories out of which the model could not learn to hit the ball at all for 6 ball trajectories (see last example ball trajectory in **Supplementary Figure 2D**). For 6 of the remaining 40 ball trajectories, the model’s performance (hit to miss ratio) primarily kept improving during the first 80% of the repeats (see red dots above 0.8 in the right panel of **Supplementary Figure 2E** and first and fourth example ball trajectories in **Supplementary Figure 2D**), whereas for the other 18 ball trajectories, the models’ performance primarily kept declining during the last 80% of the repeats (see red dots below 0.2 in the right panel of **Supplementary Figure 2E** and second example ball trajectory in **Supplementary Figure 2D**). We found that for the 16 ball trajectories, the model first learned to hit the ball and then unlearned or kept forgetting as is indicated by red dots between 0.2 and 0.8 in the right panel of **Supplementary Figure 2E** and the third example ball trajectory shown in **Supplementary Figure 2D**. The model displayed an optimal performance for a ball trajectory where the peak hit to miss ratio was 7 and the minimum value for hit to miss ratio (not shown). The best sustained performance was observed for the ball trajectory for which the hit to miss ratio remained around 0.8 for about 80 repeats (see fourth ball trajectory in **Supplementary Figure 2D**). This ball trajectory was repeated most frequently over 120 times during the training episodes. One of the reasons for a large variance in performance during training could be the intrinsic noise in the circuit which was intentionally kept higher to allow the circuit to explore action space to its full capacity. Ideally, the drive by the noise processes should decrease with learning to enable motor neurons to take actions based on the sensory inputs and the sensory-motor associations the model learned during training. However, we have not tested the use of adaptive noise in this work. The other reason for large variance in performance could simply be the fact that the performance presented in **Supplementary Figure 2** is during learning which is an extremely dynamic situation. Since each training episode (or controls) was simulated for 500 sec, using larger action timesteps of 50 ms (compared to 20 ms for feedforward model) reduced the repeats of each ball trajectory which would have caused a large variance in performance.


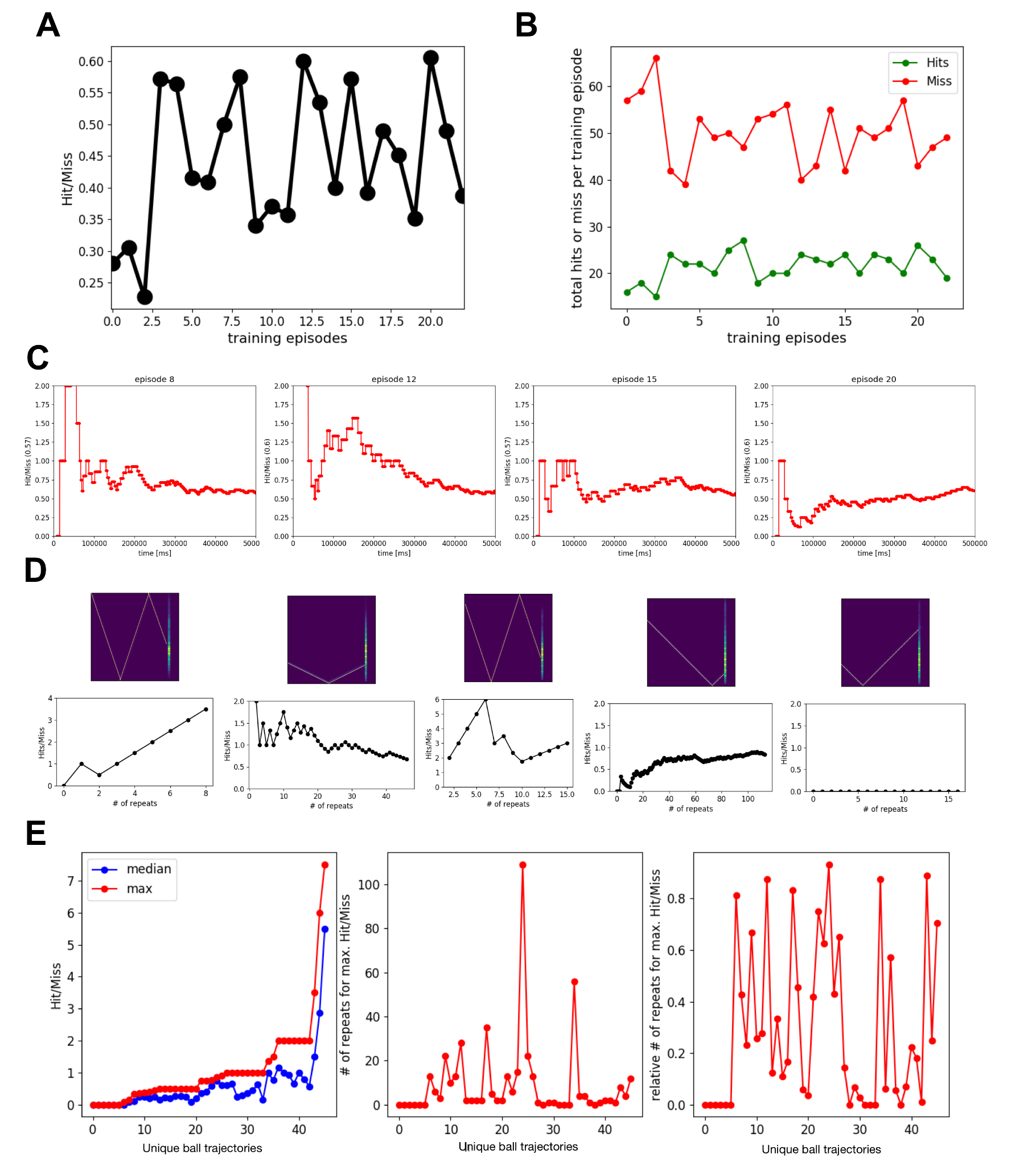


**Supplemental Figure 2**. Performance of the recurrent model with *retrograde targeted RL*. **A)** cumulative performance of 23 training episodes. **B)** Cumulative Hits and Misses of 40 training episodes. **C)** Temporal evolution of performance during training episodes 8, 12, 15, 20 (selected arbitrarily). **D)** Examples of ball trajectories with model’s learning performance shown for different modes of learning. First example shows a ball trajectory for which the model kept learning. Second example shows a ball trajectory for which the model performed well at the beginning of the training and then kept forgetting as shown by decrease in hit to miss ratio. In the third example, the model learned in the beginning and then forgot and then again started learning. Fourth example showed sustained performance as the model’s ability to hit the ball plateaued and remained constant for 80 repeats. In the fifth example, the model was unable to learn how to hit the ball for this ball trajectory. **E)** The model’s performance for different ball trajectories: The left panel shows the median and maximum Hit/Miss values during repeated occurrences of the unique ball trajectories. The middle panel shows the number of repeats of the unique ball trajectories at which the model showed peak performance. The right panel shows the relative number of repeats of the unique ball trajectories at which the model showed the peak performance. This indicates that for some ball trajectories (# 30-32), the model performed best without any training and the training only reduced the performance of the model. For some ball trajectories (seq # 0-5), the model could not learn to hit the ball. This also shows that for some ball trajectories (see the seqs with relative # of repeats for max. Hit/Miss values between 0.2 and 0.8), the model first learns to hit the ball and then forgets, whereas for a few ball trajectories (see the seqs with relative # of repeats for max. Hit/Miss values 0.8 or above), the model did not forget how to hit the ball until the end of all training episodes.


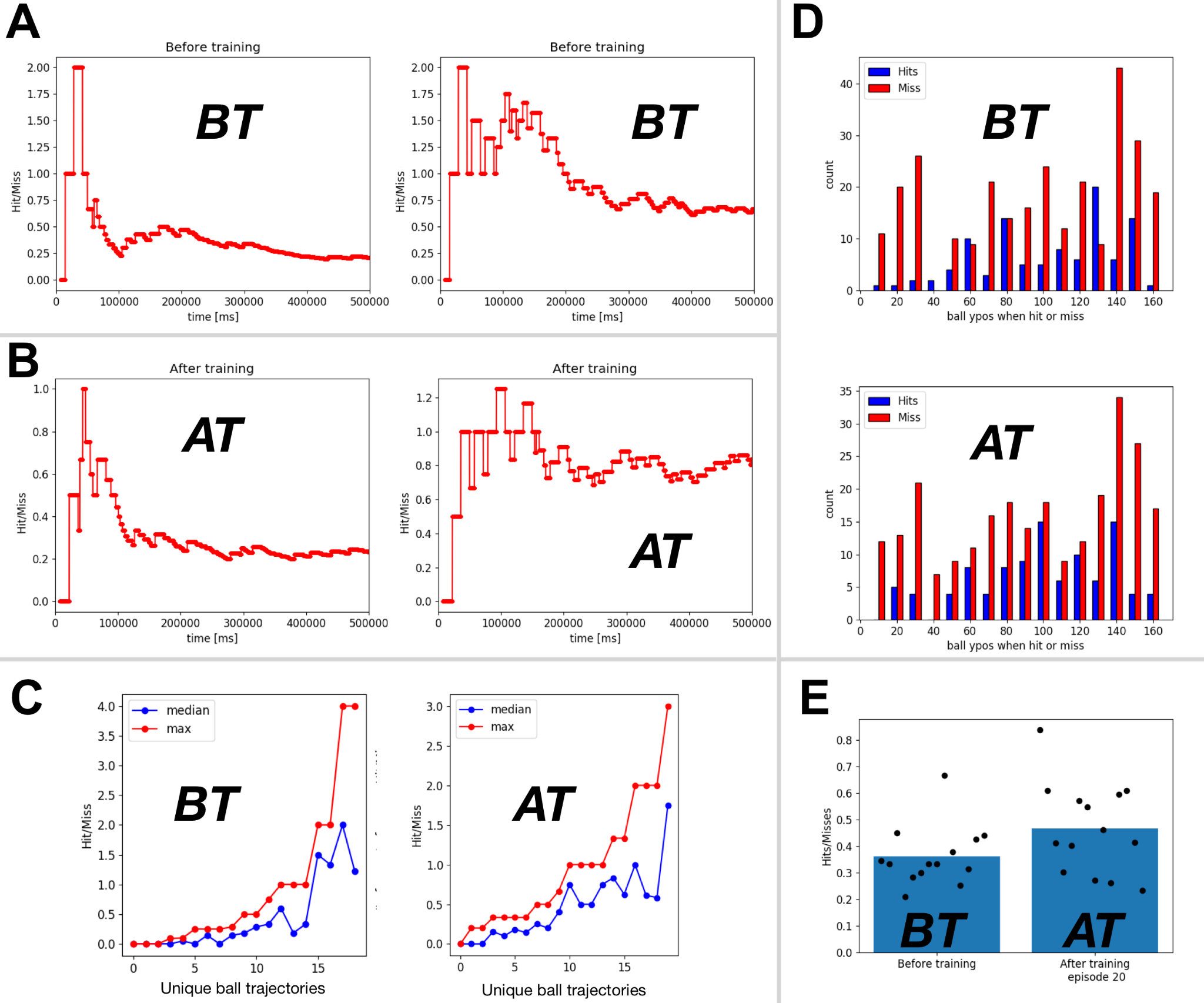


**Supplementary Figure 3.** **The recurrent spiking neuronal network model using retrograde targeted RL showed variable performance after learning.** **A)** Temporal evolution of the performance of two example simulations using different initial positions of the racket and the ball and initial weights for synaptic connections. **B)** same as in A using weights after training episode 20 for synaptic connections. **C)** Summary of the peak and the median performance for all different ball trajectories for the model before training and using weights after training episode 20. **D)**. The histogram of ‘Hits’ and ‘Misses’ against the ball’s vertical position (ypos) when crossing the racket for the model before and after training **E)** The bar plot shows the mean (n=14; filled circles) performance (Hit/Miss) of the model before training (using initial weights), after training episode 20. For the performance comparison, we ran 500 sec simulations using 14 different initial positions of the racket and the ball.

### 
